# Expanding the genetic spectrum of giant axonal neuropathy: Two novel variants in Iranian families

**DOI:** 10.1002/mgg3.2159

**Published:** 2023-03-03

**Authors:** Mahmoud Reza Ashrafi, Ali Zare Dehnavi, Ali Reza Tavasoli, Morteza Heidari, Masoud Ghahvechi Akbari, Ali Reza Ronagh, Mohammad Ghafouri, Nejat Mahdieh, Pouria Mohammadi, Zahra Rezaei

**Affiliations:** ^1^ Ataxia Clinic, Pediatric Neurology Division, Children's Medical Center, Pediatrics Center of Excellence Tehran University of Medical Sciences Tehran Iran; ^2^ Department of Paediatrics, Division of Paediatric Neurology, Growth and Development Research Center, Children's Medical Centre, Paediatrics Centre of Excellence Tehran University of Medical Sciences Tehran Iran; ^3^ Myelin Disorders Clinic, Pediatric Neurology Division, Children's Medical Center, Pediatrics Center of Excellence Tehran University of Medical Sciences Tehran Iran; ^4^ Jefferson Institute of Molecular Medicine Thomas Jefferson University Philadelphia Pennsylvania USA; ^5^ Physical Medicine and Rehabilitation department, Children's Medical Center Tehran University of Medical Sciences Tehran Iran; ^6^ Pediatric Neurology Department Alborz University of Medical Sciences Karaj Iran; ^7^ Genetic Research Center, Rajaei Cardiovascular Medical and Research Center Iran University of Medical Sciences Tehran Iran; ^8^ Department of Medical Genetics, Faculty of Medical Sciences Tarbiat Modares University Tehran Iran

**Keywords:** Giant Axonal Neuropathy, gigaxonin, WES

## Abstract

**Background:**

Giant axonal neuropathy (GAN) is a progressive childhood hereditary polyneuropathy that affects both the peripheral and central nervous systems. Disease‐causing variants in the gigaxonin gene (*GAN*) cause autosomal recessive giant axonal neuropathy. Facial weakness, nystagmus, scoliosis, kinky or curly hair, pyramidal and cerebellar signs, and sensory and motor axonal neuropathy are the main symptoms of this disorder. Here, we report two novel variants in the *GAN* gene from two unrelated Iranian families.

**Methods:**

Clinical and imaging data of patients were recorded and evaluated, retrospectively. Whole‐exome sequencing (WES) was undertaken in order to detect disease‐causing variants in participants. Confirmation of a causative variant in all three patients and their parents was carried out using Sanger sequencing and segregation analysis. In addition, for comparing to our cases, we reviewed all relevant clinical data of previously published cases of GAN between the years 2013–2020.

**Results:**

Three patients from two unrelated families were included. Using WES, we identified a novel nonsense variant [NM_022041.3:c.1162del (p.Leu388Ter)], in a 7‐year‐old boy of family 1, and a likely pathogenic missense variant [NM_022041.3:c.370T>A (p.Phe124Ile)], in two affected siblings of the family 2. Clinical examination revealed typical features of GAN‐1 in all three patients, including walking difficulties, ataxic gait, kinky hair, sensory‐motor polyneuropathy, and nonspecific neuroimaging abnormalities. Review of 63 previously reported cases of GAN indicated unique kinky hair, gait problem, hyporeflexia/areflexia, and sensory impairment were the most commonly reported clinical features.

**Conclusions:**

One homozygous nonsense variant and one homozygous missense variant in the *GAN* gene were discovered for the first time in two unrelated Iranian families that expand the mutation spectrum of GAN. Imaging findings are nonspecific, but the electrophysiological study in addition to history is helpful to achieve the diagnosis. The molecular test confirms the diagnosis.

## Graphical abstract

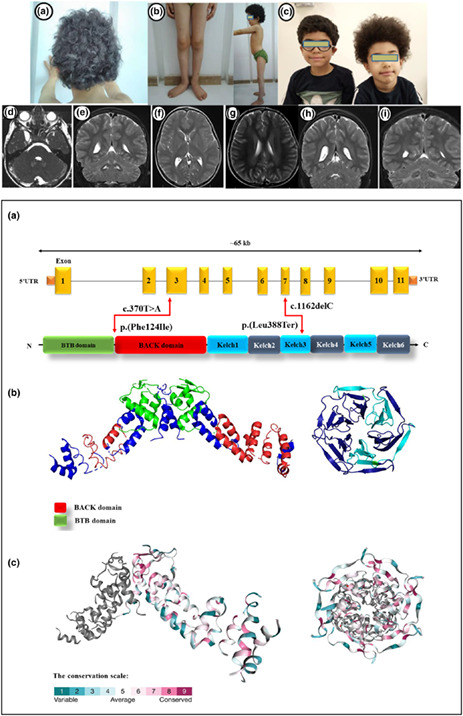

## 1 INTRODUCTION

Giant axonal neuropathy (GAN) is a rare neurodegenerative disorder that is characterized by nonspecific and heterogeneous clinical manifestations encompasses involving both the peripheral and central nervous systems. Gait and balance problems, decreased or absent deep tendon reflexes (DTRs), and cerebellar impairment are the most common neurological symptoms. Nonneurological manifestations are including endocrine, orthopedic, as well as gastrointestinal tract issues. Unique kinky or curly hair is another distinctive clinical feature that develops as a result of keratin filament disruption; however, it is not an essential diagnostic feature. (Abu‐Rashid et al., 2013; Hentati et al., 2013; Normendez‐Martínez et al., 2018; Wang et al., 2014). The onset of disease could be varied though the majority of patients, it usually presents at 3–4 years of age (Akagi et al., 2012; Johnson‐Kerner et al., 2014).

GAN‐1 (OMIM: 256850) is an autosomal recessive neurodegenerative disorder that was first reported by Asbury and Berg in the year 1972. Then, gigaxonin (*GAN*; OMIM: 605379), the responsible gene for the disorder, was discovered on chromosome 16q24.1 in the year 2000. The *GAN* encodes gigaxonin protein, which is responsible for protein degradation through the ubiquitin‐proteasome system (Akagi et al., 2012). GAN‐2 (OMIM:610100) is an autosomal‐dominant neurodegenerative disorder due to mutation in *DCAF8* gene (OMIM:615820) that is located on chromosome 1q23. It is characterized by axonal neuropathy, muscular weakness, and atrophy during the second decade of life, as well as cardiomyopathy in severe cases (Akagi et al., 2012; Klein et al., 2014).

This neurodegenerative disorder was historically named based on pathologic samples showing intermediated filaments (IF) accumulation and axonal loss in neuronal cells. Magnetic resonance imaging (MRI) reveals several nonspecific abnormal findings such as generalized brain atrophy as well as abnormal signals in cerebellar white matter, middle and superior cerebellar peduncles, posteromedial part of thalamus, internal and external capsules, bilateral periventricular and subcortical white matter, brain stem, and dentate nucleus. Imaging may be normal in the early phases of the disease; however, abnormal findings appear as the disease progresses (Abu‐Rashid et al., 2013; Hentati et al., 2013; Wang et al., 2014).

Before the molecular tests era, the diagnosis of GAN was made based on pathological findings following sural nerve biopsy that usually reveals giant axonal swelling, fiber loss, and neurodegeneration. However, giant axon is not pathognomonic of GAN diagnosis (Abu‐Rashid et al., 2013).

On the Human Gene Mutation Database (HGMD) (http://www.hgmd.cf.ac.uk), a total of 83 variants have been reported thus far. In this study, we aimed to report three genetically confirmed cases with GAN‐1, while presenting two novel variants in the gigaxonin gene. We also reviewed all clinical, imaging, and genetic data of previously reported cases from 2013 to 2020.

## 2 METHODS

### 2.1 Study subjects and ethical considerations

An informed consent was obtained from all participant families. This study was approved by the local ethics committee of the National Institute for Medical Research Development of Iran under the code of IR.NIMAD.REC.1397.508 and has therefore been performed in accordance with the ethical standards laid down in the 1964 Declaration of Helsinki and its later amendments.

### 2.2 Blood sampling and DNA isolation

After collecting 10 mL of peripheral blood from the patients and their parents, genomic DNA was extracted using the standard salting‐out method (Kalousová et al., 2017). We used agarose gel electrophoresis and spectrophotometry absorbance reading at 260 nm to evaluate the concentration and quality of the extracted DNA. Then about 3 μg of extracted DNA from the patients was used to perform Whole‐exome Sequencing.

### 2.3 Whole‐exome sequencing and bioinformatics analysis

The Agilent SureSelectV6 kit was used to enrich the whole human exome. The Agilent Sureselect Kit uses capture primers along with magnetic beads to target and capture exons. WES was performed by using the Illumina NovaSeq 6000 platform to generate 8 Gb of sequencing data with an average coverage of about 100x.

The WES raw data (FastQ file) was analyzed by bioinformatics tools based on similar studies (Mohammadi, Daneshmand, et al., 2021; Mohammadi, Heidari, et al., 2021; Mohammadi, Salehi Siavashani, et al., 2021). The FastQC was used to perform quality control (QC) of reads based on GC content and Phred value (Gaur & Chaturvedi, 2017). We used the Burrows–Wheeler aligner (BWA) aligning tool to align the sequence reads to the (GRCh38/hg38) human reference genome (Abuín et al., 2015). Postalignment QC was performed by the Picard command‐line tools (Choi et al., 2016). VarScan mpileup2 and Genome Analysis Toolkit Haplotypecaller (GATK HC) were used for calling single nucleotide (SNV) and insertion/deletion (indel) variants, and then variant call format (VCF) annotation was performed by Ensembl variant effect predictor (VEP) (McLaren et al., 2016; Ren et al., 2018).

We filtered the annotated VCF with R programming command‐line software. First, we removed variants with a minor allele frequency (MAF) >1% based on genomic databases such as gnomAD, ESP, ExAC, 1000 Genome project, and Iranome. In the next step, we filtered the remaining variants based on their consequences, and we focused on coding variants. Then, based on the human phenotype ontology (HPO) terms, we removed variants that were not related to the phenotypes. The considered HPO nomenclatures include the following terms: delayed ability to walk (HP: 0031936), curly hair (HP: 0002212), reduced deep tendon reflexes (HP: 0001315), short stature (HP: 0004322), scoliosis (HP: 0002650), and gait ataxia (HP: 0002066). Finally, the remaining variants were classified according to the American College of Medical Genetics and Genomics (ACMG) guideline (Richards et al., 2015).

### 2.4 Mutation confirmation and segregation analysis

Confirmation of identified variants and segregation analysis in the family were performed by Sanger sequencing using an Applied Biosystems 3130 Genetic Analyzer. We designed the primers with PrimerQuest and PCR amplification for the region harboring the identified variants was performed. We utilized Codoncode aligner software for the analysis of Sanger sequencing results. Functional domains and regions of the GAN protein were identified by UniProt and ConSurf servers. Also, PYMOL software was used in order to design a 3D structural model of the protein (Ashkenazy et al., 2016; DeLano, 2002).

### 2.5 Review literature

A thorough literature search was undertaken to tracking down patients with *GAN* mutations from the years 2013–2020. Numerous databases, including PubMed, JohnWiley, and Springer, were investigated in order to extract clinical, imaging, and genetic data of reported cases of GAN. All data are summarized in Table 2.

## 3 RESULTS

### 3.1 Clinical presentation

#### 3.1.1 Family 1: One patient

A 7‐year‐old boy was referred to the ataxia clinic, Children's Medical Center, Tehran, Iran due to a gait problem. He was born through a caesarian section following an uneventful term pregnancy. His birth head circumference, weight, and height were all within the normal range. He was the first child of a consanguineous family, and his sister was a 6‐year‐old healthy girl. No similar history was noted in the family. He began to walk unsteadily at the age of 2 years and this worsened over time. Therefore, his parents are concerned about his motor development. Cognition and speech milestones were achieved normally.

**FIGURE 1:**
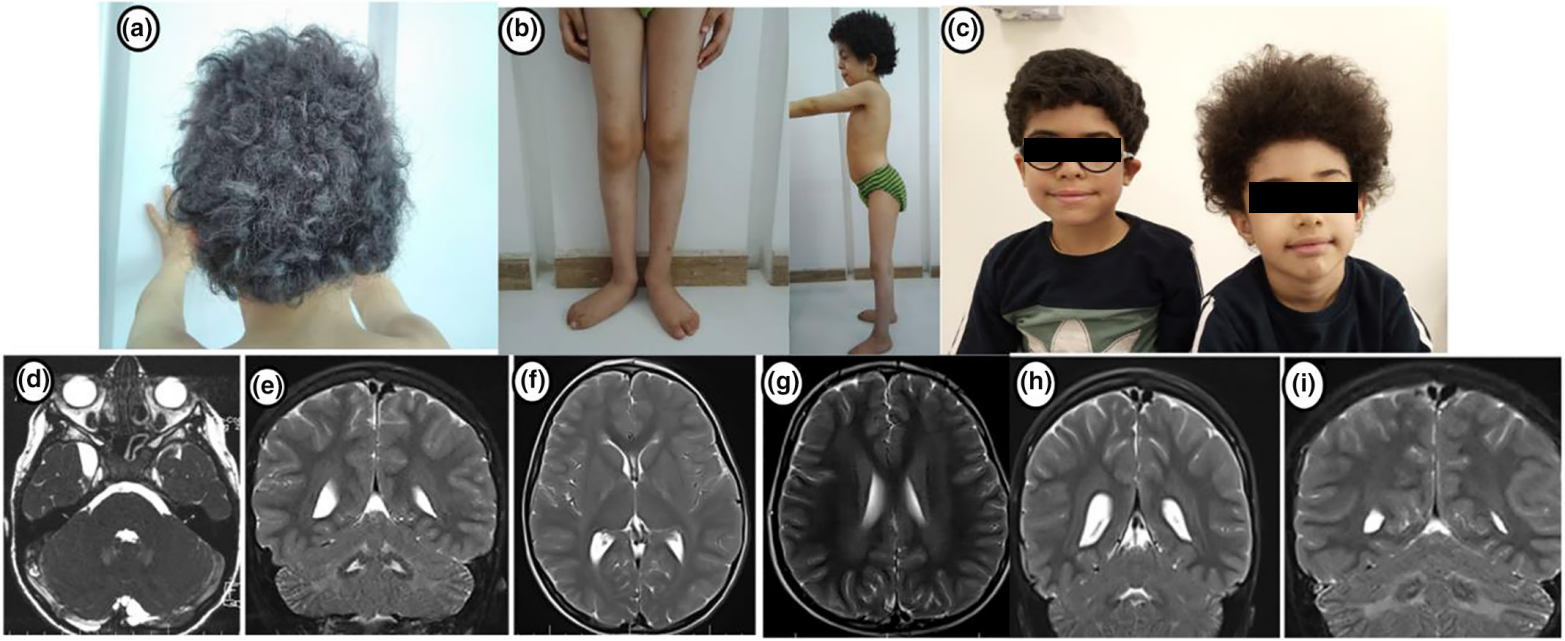
Curly hair, genu valgus, Pes planovalgus, and lordosis in patient 1, and kinky hair of patients 2 and 3 are shown in (a–c), respectively. Axial and coronal *T*_2_W sequences of the brain MRI of patient 1 show a right temporal arachnoid cyst, peridentate white matter signal changes, internal capsule involvement, and supratentorial white matter signal changes (d–g). Mild ventriculomegaly, supratentorial, and peridentate white matter signal changes are depicted in coronal T2 brain MRI images of patient 2 (h,i).

Upon examination at 7 years old, he was completely alert and his speech was normal with no dysarthria. His school function was normal as well. His weight and height were compatible with the 10th and 5th percentiles for age, respectively. Irrespective of obvious unsteadiness especially in running and turning, he was able to walk independently. His curly hair was a remarkable finding that was not observed in his parents or sibling (Figure 1a). On neurologic examination, biceps deep tendon reflexes were symmetrically normal; however, lower extremities reflexes were absent as well as symmetrical bilateral downward plantar reflexes. Neurologic examination was unremarkable for spasticity, paresis, muscular atrophy or fasciculation, myoclonus, stiffness, or dystonia. The sensory evaluation demonstrated vibration and position impairment in lower limbs without any sensory level. An ophthalmologic examination revealed a corneal ulcer in addition to photosensitivity. Additionally, significant lordosis, scoliosis, genu valgus, and pes plano valgus were found on the musculoskeletal examination (Figure 1b).

In the electrophysiological evaluation, a chronic sensorimotor distal polyneuropathy with an axonal feature of the lower limbs was reported. Brain MRI revealed bilateral nonspecific signal changes of supra‐ and infratentorial white matter, dentate nucleus, peridentate white matter, and internal capsule as well as right temporal arachnoid cyst (Figure 1d–g). Basic metabolic laboratory tests were also unremarkable. Next‐Generation Sequencing was performed to achieve a molecular diagnosis.

#### 3.1.2 Family 2: Two patients

Patients 2 and 3, 7‐ and 4‐year‐old boys, respectively, were the first and the second children of a consanguineous and healthy parent. Both patients were born through an uneventful pregnancy following a normal vaginal delivery. Their birth head circumference, weight, and height were all within the normal range. Following initial normal development, neurological symptoms, including gait disturbance and walking difficulty, started in patient 2 when he was 2 years old. Several medical evaluations were not conclusive. He was referred to the ataxia clinic when he was 6 years old. At the first visit, his weight and height were compatible with the 10th and 25th percentiles for age, respectively. Speech and cognition milestones were normal. However, he had ataxic spastic gait. He was able to walk without support. The neurologic examination revealed contractures in the lower limbs' joints, absent deep tendon reflexes, and muscle atrophy. No paresis, fasciculation, myoclonus, rigidity, chorea, tremor, or dystonia was detected. Sensory evaluation was unremarkable as well. In addition, kinky curly hair and horizontal nystagmus were noticed on examination (Figure 1c). Chronic axonal‐type sensorimotor polyneuropathy of lower limbs was displayed in electrophysiological study. Mild dilation of the lateral ventricles and nonspecific signal changes of supra tentorial and peridentate white matter were detected on brain MRI (Figure 1h,i).

The younger brother had normal growth and development during the first year of life. Then after, motor development impairment was prominently led to a gait problem. At the age of 3, he was seen in the ataxia clinic for the first time. His weight and height were compatible with the 3rd and 25th percentiles for age, respectively. Intellectual and language milestones were normal. Irrespective of his ability to walk independently, ataxic gait and frequent falls especially in turning were noticed. The Neurologic examination revealed hypotonia, muscular atrophy, and reduced lower limb DTRs. Mild Scoliosis and multiple contractures of both upper and lower limbs caused him a few problems in doing daily activities. Similar to his older brother, tightly curled hair was also noted. Other parts of his neurologic examination including sensory and ophthalmologic evaluations were unremarkable. Basic metabolic tests were normal. The neurophysiologic study revealed lower limbs chronic axonal‐type sensorimotor polyneuropathy. By considering the similarity between the clinical course of these two siblings, a familial neurodegenerative disorder was suspected. Next‐Generation Sequencing was performed to achieve a molecular diagnosis. Brain MRI for patient 2 was nonspecific but was not performed for the younger brother.

### 3.2 Exome sequencing results and segregation analysis

In family 1, WES identified a novel homozygous nonsense variant in *GAN* (OMIM: 605379), [NM_022041.3: c.1162delC; p.Leu388Ter], resulting in a premature stop codon at position 388. This variant was classified as pathogenic according to the ACMG variant classification. In family 2, a novel homozygous missense variant, [NM_022041.3: c.370T>A; p.Phe124Ile], was identified in *GAN*, which caused an amino acid change from Phe to Ile at position 124. This variant was classified as likely pathogenic according to the ACMG variant classification. These variants were not reported in the gnomAD, ExAC, and Iranome databases. The identified variants were confirmed by Sanger sequencing and segregated with the disease in the families (Table 1).

**TABLE 1:** Characteristics of the identified sequence variants in our patients based on online databases.

Patient	Gene	Variant coordinates	In silico parameters	Allele frequencies^a^	Type and classification^b^	Zygosity	ACMG rules	Cadd Phred
Case 1	*GAN1*	Chr16(hg38):g 81,363,869 NM_022041.3 c.1162delC (p.Leu388Ter) Exon 7/11	MutationTaster: NA FATHMM‐MKL: NA EIGEN: NA	gnomAD: NA ExAC: NA Iranome: NA	Stop gain Pathogenic (Class 1)	Homozygous	PVS1 PM2 PP4	NA
Case 2 and Case 3	*GAN1*	Chr16(hg38):g 81,354,492 NM_022041.3: c.370T>A (p.Phe124Ile) Exon 3/11	MutationTaster: Disease causing FATHMM‐MKL: Damaging EIGEN: Pathogenic	gnomAD: NA ExAC: NA Iranome: NA	Missense Likely pathogenic (class 2)	Homozygous	PM2 PP2 PP3 PP4	28.5

^a^ Genome Aggregation Database (gnomAD) Genome version:3.0, Exome Aggregation Consortium (ExAC) version:1.0 and Iranome.

^b^ Variant classifcation is based on ACMG recommendations: Class 1: Pathogenic, Class 2: Likely pathogenic, Class 3: Variant of uncertain significance (VUS), Class 4: Likely benign, Class 5: Benign.

### 3.3 Clinical findings of reported GAN patients

Table 2 compares the clinical, imaging, and electrodiagnostic characteristics of patients with GAN reported between 2013 and 2020. Unusual kinky hair (82%), gait problem (80%), and areflexia/hyporeflexia (66%) were the most prevalent reported symptoms. Reported neuroimaging findings varied from normal imaging to nonspecific signal changes in cerebral white matter, brain stem, dentate nucleus, and internal capsule. Besides, sensorimotor (axonal/demyelinating) polyneuropathy was the most frequent finding in electrophysiological studies.

**TABLE 2:** Clinical and Paraclinical data of reported patients with GAN from 2013 to 2020.

Study/year	Number of cases	The most common clinical manifestations							Imaging findings	Electrodiagnostic findings
Current Study/2021	Case 1	Motor dysfunction	Sensory dysfunction	Cerebellar dysfunction	Characteristic hair feature	Musculoskeletal/orthopedic problem	Cranial nerve involvements	Additional problems	Supra‐ and infratentorial white matter signal changes, Ventriculomegaly	Chronic sensorimotor distal polyneuropathy with axonal feature
		Delayed motor milestones, reduced reflexes	Impaired sensation	Ataxic gait	Curly hair	Muscular atrophy, Joint contracture, short stature, Spine Deformity, Pes planovulgus, Genu valgus	No	Photosensitivity, Transient corneal ulcer		
	Case 2	Gait problem, Spastic gait, Areflexia	No	Ataxic gait	Curly hair	Muscular atrophy, Joint contracture	Ophthalmologic (Nystagmus)	No	Mild ventriculomegaly	Chronic sensorimotor polyneuropathy
	Case 3	Falling, Gait problem, Reduced reflexes, Hypotonia	No	Ataxic gait	Curly hair	Joint contracture, Scoliosis	No	No	Not available	Chronic sensorimotor polyneuropathy
Abu‐Rasid et al. (2013)	4	Delayed motor milestones (4/4), Areflexia (4/4), Spasticity (3/4)	Impaired sensation (4/4)	Truncal ataxia (2/4)	Kinky hair (4/4)	Foot deformity (3/4), Muscular atrophy (3/4), Spine deformity (4/4), Short stature (4/4)	No	Precocious puberty (4/4)	Supratentorial white matter signal changes, Posterior limb of internal capsule, Dentate nuclei, Middle cerebellar peduncle	Sensorimotor demyelinating neuropathy
Roth et al. (2014)	13	Gait problem/Delayed motor milestones (13/13)	Not available	Not available	Curly hair (11/13)	Not available	Ophthalmologic (12/13), Bulbar (10/13)	Gastro‐ intestinal (12/13), Cognitive (2/13)	Not available	Not available
Wubben et al. (2013)	1	Gait problem, Areflexia	Impaired posterior column sensation	Not available	Kinky hair	Weakness, Hand deformity	Ophthalmologic, Bulbar	Respiratory, Gastrointestinal, Ophthalmologic	Not available	Not available
Kamate et al. (2014)	2	Gait problem (2/2), Delayed motor milestones (2/2), Spastic gait (2/2), Areflexia (2/2)	Not available	Cerebellar dysfunction (2/2)	Curly hair (2/2)	Foot deformity (2/2)	Ophthalmologic (2/2)	Cognitive problems (2/2)	Dentate nucleus, Internal capsule	Not available
Wang et al. (2014)	3	Gait problem (3/3), Delayed motor milestones (1/3)	Impaired sensation (3/3)	Ataxic gait (3/3), Cerebellar dysfunction (3/3)	No (3/3)	Weakness (3/3), Spine deformity (3/3)	Cranial nerve involvement (1/3)	Incontinence (2/3), Seizure (1/3), Dementia (1/3)	Brain atrophy, Supra‐ and infratentorial white matter changes, internal capsule, thalamus, brain stem, cerebellar peduncles	Chronic sensorimotor (axonal and demyelinating) polyneuropathy
Incecik et al. (2015)	8	Falling (2/8), Gait problem (4/8), Areflexia (8/8)	Not available	Cerebellar Dysfunction (5/8)	Kinky hair (8/8)	Weakness (2/8), Spine deformity (4/8), Foot deformity (2/8)	Ophthalmologic (3/8), Facial weakness (2/8)	Babinski Sign (2/8), Cognitive (3/8)	Cerebral/cerebellar atrophy, White matter signal changes	Axonal sensorimotor neuropathy
Mohammad et al. (2014)	1	Delayed motor milestone, Areflexia,	No	Not available	Curly hair	Weakness, Foot deformity, Joint hypermobility	Not available	Not available	Normal	Axonal sensorimotor neuropathy
Jain et al. (2014)	4	Hypotonia (3/4), Delayed motor milestones (4/4), Gait problem (4/4), Areflexia (4/4), Falling (2/4), Spastic gait (2/4)	Not available	Cerebellar dysfunction (4/4)	Frizzy Hair (4/4)	Foot deformity (2/4)	Ophthalmologic (2/4)	Seizure (3/4), Cognitive (2/4)	Dentate nucleus, Globus Pallidus, Internal capsule	Axonal sensorimotor polyneuropathy
Israni et al. (2014)	1	Gait problem, Quadriparesia, Hypotonia	Not available	Cerebellar dysfunction	Curly Hair	Not available	Not available	Auditory, Cognitive	White matter signal changes, Internal capsule	Sensorimotor Polyneuropathy
Vijaykumar et al. (2015)	1	Gait problem, Hypotonia, Falling, Areflexia	Not available	Cerebellar dysfunction, Ataxic gait	Curly hair	Foot deformity, Weakness	Ophthalmologic, Facial weakness	Babinski sign	Supra‐ and infratentorial white matter signal changes, Internal capsule	Axonal sensorimotor neuropathy
Miyatake et al. (2015)	1	Falling, Motor regression, Areflexia,	Impaired sensation	Ataxic gait	Frizzy hair	Spine deformity Muscular atrophy, Weakness	Ophthalmologic, Facial weakness, Bulbar dysfunction	Failure to Thrive, Cognitive, Respiratory	Supra‐ and infratentorial white matter signal changes, Basal ganglia, Thalamus, cerebellar cysts	Sensory‐motor Neuropathy
Koichihara et al. (2016)	1	Gait Problem, Hyperreflexia	Not available	No	No	Weakness, Muscle atrophy Foot deformity	No	No	Normal	Neurogenic pattern
Aharoni et al. (2016)	5	Hypo/areflexia (4/5), Gait problem (4/5), Falling (2/5)	Impaired posterior column sensation (5/5)	No	Kinky hair (1/5)	Foot deformity (5/5), Weakness (5/5), Joint contracture (2/5), Muscular atrophy (1/5)	Facial weakness (1/5), Bulbar dysfunction (1/5)	Cognitive (1/5), Babinski Sign (2/5)	White matter signal changes	Normal motor and sensory nerve conduction velocity, reduced amplitude CMAP*
Almeida et al. (2016)	1	Hypotonia, Gait problem	Not available	Not available	Curly Hair	Weakness, Tendon Shortening, Foot deformity, Joint hypermobility	Not available	Precocious Puberty, Irregular eyelash	Diffuse hypomyelination	Not available
Normendez‐Martínez et al. (2018)	1	Gait problem, Areflexia	No	Ataxic gait	Curly hair	Muscular atrophy, Weakness	Ophthalmologic	No	Not available	Axonal sensorimotor neuropathy
Garg et al. (2018)	3	Hypotonia (1/3), Gait problem (3/3), Fine motor Dysfunction (1/3), Areflexia (2/3), Delayed motor milestones (2/3)	Impaired posterior column sensation (1/3)	Cerebellar dysfunction (2/3)	Curly Hair (2/3)	Foot deformity (2/3), Spine deformity (2/3), Joint contractures (1/3), Short stature (1/3)	Facial weakness (2/3), Ophthalmologic (1/3), Bulbar dysfunction (1/3)	Babinski Sign (1/3), Seizure (1/3), Cognitive (2/3), Failure to Thrive (1/3), Dimorphisms (1/3)	Supra and infratentorial white matter signal changes, dentate nucleus, medulla, superior cerebellar peduncles, basal ganglia (Globus Pallidus), calcification	Axonal sensorimotor polyneuropathy
Cai et al. (2018)	1	Gait problems, Exercise intolerance, Areflexia,	Limb numbness, Impaired sensation	Cerebellar dysfunction, Ataxic gait	Kinky Hair	Foot deformity Muscular atrophy, Weakness, Spine deformity	Ophthalmologic	Babinski sign	Lesions around the 4th ventricle	Axonal sensorimotor neuropathy
Echaniz‐Laguna et al. (2020)	10	Areflexia (10/10), Loss of ambulation (3/10)	Not available	Ataxic gait (10/10)	Curly hair (10/10)	Joint Contracture (2/10), Weakness (7/10)	Ophthalmologic (2/10), Bulbar dysfunction (4/10)	Precocious Puberty (1/10), Respiratory (3/10), Dermatologic (1/10), Ophthalmologic (1/10)	Dentate nucleus, white matter signal changes, thalamus, brain stem	Axonal/demyelinating sensory neuropathy
Edem et al. (2019)	1	Gait problems, Falling, Areflexia	Not available	Cerebellar dysfunction, Ataxic gait	Not available	Foot deformity, Weakness, Muscular atrophy	Ophthalmologic	Pronunciation problems, Cognitive, Gastrointestinal, Dermatologic	Dentate nuclei, cerebellar peduncles, and white matter signal changes	Axonal sensorimotor polyneuropathy
Xu et al. (2020)	1	Gait Problem, Areflexia, Delayed motor milestones	Not available	Cerebellar dysfunction	Curly hair	Hip dislocation, Weakness	Not available	Respiratory	Normal	Axonal neuropathy
Total/The most common presentation	66	Gait problem (53/66, 80%) Areflexia/decreased reflexes (44/66, 66%) Delayed motor milestones (29/66, 43%)	Impaired sensation (10/55, 18%) Impaired posterior column sensation (7/55, 12%)	Ataxic gait (21/50, 42%)	Curly hair (54/65, 83%)	Weakness (29/52, 55%) Foot deformity (23/52, 44%) Spine deformity (17/52, 32%)	Ophthalmologic (29/62, 46%) Bulbar dysfunction (19/62, 30%) Facial weakness (7/62, 11%)	Cognitive (15/65, 23%) Gastrointestinal (13/65, 20%) Ophthalmologic (8/65, 12%)	White matter signal changes Dentate nucleus signals Internal capsule signals Cerebellar peduncles/basal ganglia signals	Axonal sensorimotor polyneuropathy

Abbreviation: CMAP, compound muscle action potential.

## 4 DISCUSSION

Neurofilaments (NFs), especially abundant in axons, are neuronal intermediate filaments that are considered necessary for normal axonal growth and maintenance in the development and signal transmission of aoxn (Yuan et al., 2017). Although the exact mechanism is not understood, cytoskeletal abnormalities and neurofilament network defects are followed by misfolding and abnormal protein accumulation, decreased mRNA expression levels, and disturbed mitochondrial motility response to oxidative stress which in turn could lead to neurodegenerative disorders such as Giant axonal neuropathy type 1 (Bomont et al., 2000; Buysse et al., 2010; Israeli et al., 2016; Johnson‐Kerner et al., 2014; Lowery et al., 2016; Shi et al., 2020).

As a member of the conserved BTB‐kelch (Broad Complex, Tramtrack, and Bric a Brac) superfamily, gigaxonin is a part of the ubiquitin‐proteasome system, which controls the degradation of MAP1B‐LC (Microtubule‐associated protein 1B‐light chain) and TBCB (Tubulin folding cofactor B) by the ubiquitin‐proteasome system (Ding et al., 2002; Wang et al., 2005; Zhang et al., 2005). Through interaction with MAP1B as a binding partner, gigaxonin indirectly binds to the cytoskeletal network. MAP1B is highly expressed during the developmental process of the nervous system, and postnatally, its expression decreases, significantly. As another interacting partner of gigaxonin, TBCB is a tubulin chaperone protein that binds to the alpha‐tubulin of microtubules (Wang et al., 2005).

Accumulation of MAP1B and TBCB proteins can disrupt the movement of motor proteins and may affect transport processes by altering microtubule dynamics, which leads to morphological and functional changes in the neurofilament network, distension and demyelination of axonal fibers, cell death, and subsequently neurodegenerative disorders such as giant axonal neuropathy (Allen et al., 2005; Bomont et al., 2000; Wang et al., 2005; Xu et al., 2020). Allen et al. showed that gigaxonin recruits ubiquitin‐activating enzyme E1 (UBA1) via its BTB domain and controls the degradation of MAP1B and TBCB. This function is essential for neurofilament architecture, axonal structure, and neuronal stability (Allen et al., 2005).

Homozygous and compound heterozygous mutations in the *GAN* gene are consistent with the Giant axonal neuropathy type 1, which is an autosomal recessive sensorimotor neuropathy (Kang et al., 2016). In this study, three Iranian patients from two unrelated families were described. A novel homozygous nonsense variant in *GAN* [NM_022041.3: c.1162delC; p.(Leu388Ter)] was identified in patient 1. Besides, a novel homozygous missense variant [NM_022041.3: c.370T>A; p.(Phe124Ile)] was identified in patients 2 and 3. The main clinical features in all three patients included delayed motor milestones, diminished deep tendon reflexes, impaired sensation, ataxic gait, orthopedic problems as well as unique curly hair, that were clinically compatible with GAN. Neuroimaging findings were nonspecific signal changes in supra‐ and infratentorial white matter, peridentate and internal capsule, ventriculomegaly as well as a right temporal arachnoid cyst in case 1. Although progressive cerebellar cyst has been, except for arachnoid cyst in case 1, we did not find a cerebellar cyst in available imaging (Miyatake et al., 2015; Tan et al., 2015). Chronic sensorimotor distal polyneuropathy with the axonal feature was reported in an electrodiagnostic study of our patients.

Data analysis of 55 cases of GAN was published by Guo et al. (2022) show that hypo/areflexia, muscle weakness, curly hair, and cerebellar dysfunction were the most detected clinical findings that are compatible with the clinical presentation of our cases. The most commonly reported electrodiagnostic and neuroimaging findings in GAN include sensorimotor neuropathy and nonspecific supra and infratentorial white matter signal changes, respectively. Regarding imaging and electrophysiologic findings, our patients were similar to reported features as well (Aharoni et al., 2016; Almeida et al., 2016; Garg et al., 2018; Koichihara et al., 2016; Mohammad et al., 2014). Although ocular abnormalities such as optic atrophy (Bacquet et al., 2018), retinitis pigmentosa (Kamate et al., 2014), ptosis, and nystagmus (Vijaykumar et al., 2015) are frequently reported in GAN, but only photosensitivity and the history of corneal ulcer were detected in patient 1.

**FIGURE 2:**
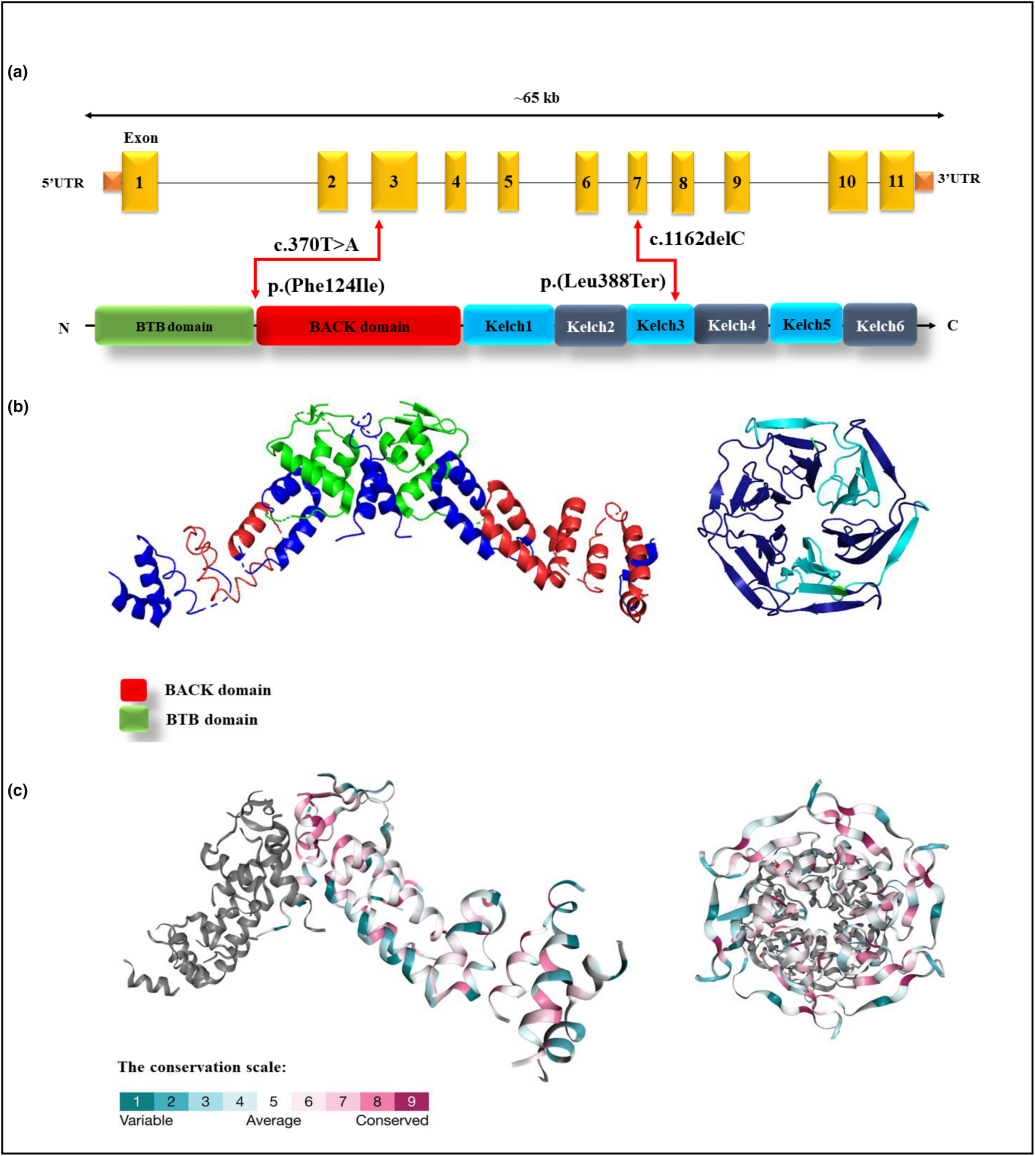
(a) The *GAN* gene contains 11 exons and spans ~65 kb. Graphical view of the gigaxonin protein updated from UniProt (UniProtKB‐Q9H2C0). The canonical isoform length of the gigaxonin protein (identifier: Q9H2C0–1) has 597 amino acids and contains N‐terminal BTB domain (residue 30–99), BACK domain (residue 134–236), C‐terminal Kelch repeats (Kelch 1: 274–326, Kelch 2: 327–374, Kelch 3: 376–421, Kelch 4: 422–468, Kelch 5: 470–522, Kelch 6: 528–574). The c.1162delC; p.(Leu388Ter) variant is in Exon 7, and Kelch 3 repeats, which creates a premature stop codon at position of 388. The c.370T>A; p.(Phe124Ile) variant is in Exon 3. (b) 3D structure of the gigaxonin protein is provided by PyMOL software. The left part shows BTB and BACK domains (green and red colors, respectively) (PDB: 3HVE), and the right part is Kelch repeats (PDB: 2XN4). (c) 3D structure of gigaxonon protein which is designed by ConSurf server database. The left figure part shows BTB and BACK domains and the right part is Kelch repeats, based on the conservation scale.

Located on chromosome 16q23.2, the *GAN* gene (OMIM# 605379), which encodes gigaxonin protein, has three protein‐coding transcripts, the biggest transcript (ENST00000648994.2) spans 15.15 kb and has 11 exons (https://asia.ensembl.org) (Ding et al., 2002). Expressed in the brain, heart, and muscle, the gigaxonin protein has one isoform, 597 amino acids, and contains N‐terminal BTB domain (residues: 30–99), which binds directly to ubiquitin‐activating enzyme E1, BAck domain (residues: 134–236) and six C‐terminal Kelch‐repeat domain which responsible for bind to the N‐terminal of TBCB and MAP1B‐LC (https://www.uniprot.org/uniprot/Q9H2C0) (Ding et al., 2002; Wang et al., 2005). Our identified variants were located in exons 3 and 7 of the *GAN* gene (Figure 2a).

GAN, as a rare neurodegenerative disorder, could present uniquely not only by involving both the upper and lower nervous systems but also by presenting with neurologic and extraneurologic manifestations (Vijaykumar et al., 2015). Indeed, kinky/curly hair, which is classified as pili canaliculi with electron microscope evaluation is not a specific diagnostic symptom (Almeida et al., 2016). Kinky hair might be an early sign and presents before other manifestations (Vijaykumar et al., 2015). Roth et al. demonstrated that the early appearance of kinky hair could be related to a more severe phenotype; therefore, a milder phenotype with straight hair might be underdiagnosed (Roth et al., 2014; Vijaykumar et al., 2015). With respect to the diversity of other reported nonspecific extraneurologic manifestations of the GAN such as bladder dysfunction, gastrointestinal manifestations, short stature, and precocious puberty, it is not possible to make a diagnosis merely based on clinical manifestations (Aharoni et al., 2016; Hoebeke et al., 2018). In addition, neuropathology finding in nerve biopsy, such as giant axons, is not only specific to GAN but also considered an invasive method (Jaffer et al., 2012). Laboratory evaluation results are also nonspecific and neuroimaging findings comprise heterogeneous and nonspecific features, that could not lead to narrowing the differential diagnosis (Aharoni et al., 2016; Almeida et al., 2016; Cai et al., 2018; Echaniz‐Laguna et al., 2020; Edem et al., 2019; Garg et al., 2018; Jain et al., 2014; Johnson‐Kerner et al., 2014; Kamate et al., 2014; Koichihara et al., 2016; Mohammad et al., 2014; Xu et al., 2020). As a result, molecular diagnostic methods, such as whole‐exome sequencing, play a key role in approaching these patients (Bacquet et al., 2018). Serial Optical Coherence Tomography (OCT) or the detection of the inclusion body inside the epithelial cells of the ocular lens could be considered advanced monitoring and diagnostic tools that need more investigation (Armao et al., 2019; Bacquet et al., 2018).

The current therapeutic approach is focused on supportive and symptomatic management as well as rehabilitation strategies (Mussche et al., 2013). Recent trials with gene therapy and viral vector application are promising that can be considered new emerging therapies in GAN (Bailey et al., 2018; Mussche et al., 2013). Last but not the least, GAN should be considered a clinical differential diagnosis in those patients who manifest neurodegeneration, gait problem, axonal sensorimotor neuropathy, simultaneous peripheral, and central nervous system involvement, with or without kinky/curly hair.

## AUTHOR CONTRIBUTIONS

MRA designed and supervised the study. ZR, PM, and ART were the major contributors to writing the manuscript. ZR, MH, ART, and AZD interpreted clinical data. NM and PM contributed to genetic analyses. MGA interpreted the electrodiagnostic data. All authors read and approved the final manuscript.

## FUNDING INFORMATION

This study was granted by NIMAD under proposal no. 971846.

## CONFLICT OF INTEREST STATEMENT

There is no conflict of interest.

## DECLARATION

Participants' families agreed on the anonymous publication of patients' clinical information and their relevant data.

## Data Availability

Human variants and pertinent phenotypes have been reported to ClinVar (Submission ID: SUB8899583). All clinical data generated or analyzed during the study are included in this published article and its supplementary information files.
